# The complete chloroplast genome of *Chimonobambusa hejiangensis*

**DOI:** 10.1080/23802359.2021.1930599

**Published:** 2021-06-03

**Authors:** Yun-Ke Liu, Wei Liu, Qian-Gang Xiao, Zhen Zeng, Li Zhuang, Ting-Hao Zhang, Jun Zhang, Qian-Ying Huang, Bao-Xin Wang

**Affiliations:** Chengdu Academy of Agricultural and Forestry Sciences, Chengdu, Sichuan, China

**Keywords:** *Chimonobambusa hejiangensis*, chloroplast genome, phylogenetic analysis

## Abstract

*Chimonobambusa hejiangensis* is of the unique edible bamboo specie of high quality in China. We studied the complete chloroplast(cp) genome of *C. purpurea* in this study. The cp genome of *C. hejiangensis* (GenBank accession: MW186792) was 138,911 bp in length, including a large single-copy (LSC) region of 82,498 bp, a small single-copy (SSC) region of 12,743 bp and a pair of inverted repeated (IR) regions of 21,835 bp. The genome contained 133 genes, including 86 protein-coding genes, 39 tRNA genes, and 8 rRNA genes. Based on 39 cp genomes, we used the phylogenetic analysis to build phylogenetic tree, indicating that *C. hejiangensis* is closely related to *C. tumidissinoda*. Also, the phylogenetic relationship of lineages might be (Hsuehochloa + (((Shibataea clade + Arundinaria clade) + Indocalamus wilsonii) + ((Bergbambos + Indocalamus) + (((African alpine bamboos + Gaoligongshania) + (Chimonocalamus + Kuruna))+(Thamnocalamus + Phyllostachys clade))))). It could be devoted to phylogenetic analysis of Arundinarieae.

*Chimonobambusa hejiangensis* C. D. Chu & C. S. Chao is one of the unique edible bamboo species of high quality in China, which is only natural distributed in Sichuan and Guizhou Provinces (Li and Stapleton 2006; Ma et al. 2016; Wang et al. 2019). With aerial roots, high ornamental value, crispy and delicious bamboo shoots and high nutritional value, *C. hejiangensis* is deeply loved by consumers all over China and Southeast Asia, and its economic benefits are very significant (Wang et al. 2016). In this study, we report and characterize the chloroplast genome of *C. hejiangensis*. We reconstruct a phylogenetic tree to reveal the relationship and provide useful information for further study of *C. hejiangensis*.

The materials of *C. hejiangensis* were collected from Chengdu City, Sichuan Province, China (N30°40′49″, E103°43′53″) on 10 June 2020. The voucher specimens were deposited at the Herbarium of Chengdu Academy of Agricultural and Forestry Sciences (http://www.cdnky.com) under the voucher number LYK20200610-05. The chloroplast DNA was extracted from fresh leaves of an individual of *C. hejiangensis*. Total DNA was used to generate libraries with an average insert size of 400 bp, sequenced using the Illumina HiSeq platform, generating 150 bp paired-end reads. Ten million high-quality reads were mapped to the published *C. tumidissinoda* chloroplast genomes as references using MUMmer v3.1 (Kurtz et al. 2004). We used A5-miseq v20150522 (Coil et al. 2015) and SPAdes v3.9.0 (Bankevich et al. 2012) to assemble these reads into complete chloroplast genomes. The assembled chloroplast genome sequence was annotated using the online Geseq web server (https://chlorobox.mpimp-golm.mpg.de/geseq.html).

The complete plastid genome sequence of *C. hejiangensis* (GenBank accession: MW186792) was 138,911 bp in length, including a large single-copy (LSC) region of 82,498 bp, a small single-copy (SSC) region of 12,743 bp and a pair of inverted repeated (IR) regions of 21,835 bp. The complete chloroplast genome contained 133 genes, including 86 protein-coding genes, 39 tRNA genes and 8 rRNA genes. The complete genome GC content was 38.92%, and the corresponding values of the LSC, SSC and IR were 37.01%, 33.20% and 44.21%, respectively.

The maximum-likelihood phylogenetic tree was constructed based on 39 complete chloroplast genomes of Bambusoideae species, and *Miscanthus transmorrisonensis* (Panicoideae) as outgroup. All of them were downloaded from NCBI GenBank. The sequences were aligned using MAFFT v7.037 (Katoh and Standley 2013), and the phylogenetic tree constructed using IQ-TREE Multicore version 1.6.12 (Gao et al. 2018). Based on the phylogenetic tree, we can see that *C. hejiangensis* has a close relationship with *C. tumidissinoda*. Also, the results supported that *Gelidocalamus tessellatus* belongs to *Shibataea* clade and the phylogenetic relationship of lineages might be (*Hsuehochloa* + (((*Shibataea* clade + *Arundinaria* clade) +*Indocalamus wilsonii*) + ((Bergbambos + *Indocalamus*) + (((African alpine bamboos+*Gaoligongshania*)+(*Chimonocalamus* + *Kuruna*))+(*Thamnocalamus+Phyllostachys* clade))))) (Ma et al. 2014, 2017; Zhang et al. 2020). It could be devoted to the phylogenetic analysis of Arundinarieae (Figure 1).

**Figure 1.**
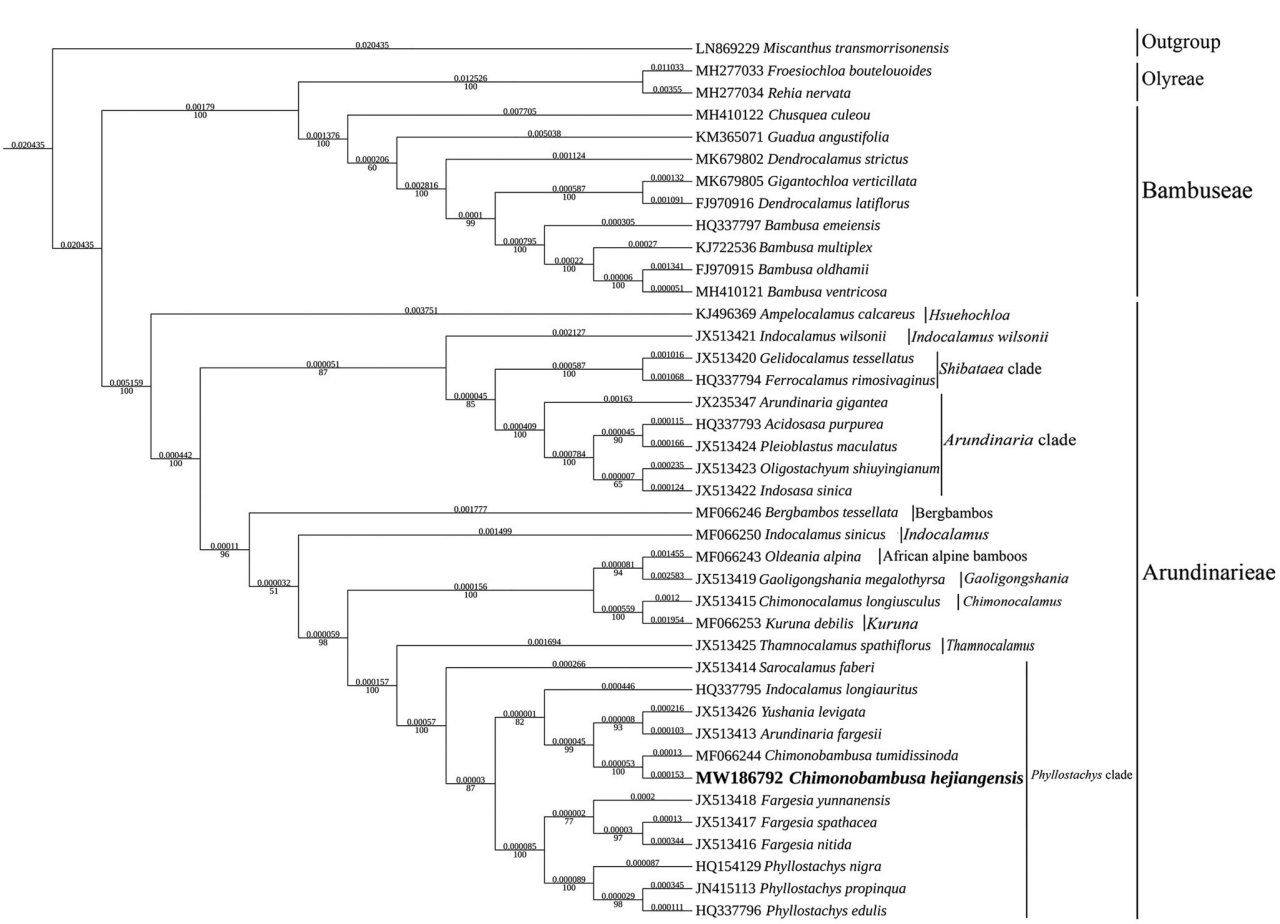
Maximum-likelihood phylogenetic analysis of 39 species of Bambusoideae and *Miscanthus transmorrisonensis* (Panicoideae) as outgroup based on plastid genome sequences by IQ-TREE multicore version 1.6.12 under GTR + R6 model for 5000 ultrafast bootstraps. Branch lengths (above) and bootstrap values (below) were indicated around nodes. GenBank accession numbers of each species were listed in the phylogenetic tree.

## Data Availability

The genome sequence data that support the findings of this study are openly available in GenBank of NCBI at (https://www.ncbi.nlm.nih.gov/) under the accession No. MW186792. The associated “BioProject”, “SRA”, and “BioSample” numbers are PRJNA684970, SRR13258744, and SAMN17073575, respectively.
